# Epidemiological and Clinical Features of Enterotoxigenic *Escherichia coli* (ETEC) Diarrhea in an Urban Slum in Dhaka, Bangladesh

**DOI:** 10.1093/ofid/ofaf375

**Published:** 2025-06-30

**Authors:** Fahima Chowdhury, Md Taufiqul Islam, Faisal Ahmmed, Afroza Akter, Martin Bundi Mwebia, Justin Im, Natasha Y Rickett, Cecilia Kathure Mbae, Asma Binte Aziz, Beatrice Ongadi, Farhana Khanam, Ashraful Islam Khan, Md Golam Firoj, Sadia Isfat Ara Rahman, Se Eun Park, Kassa Haile, Moses Mwangi, Benjamin Ngugi, Meseret Gebre Behute, Kelvin Kering, Suneth Agampodi, Suman Kanungo, K Zaman, Samuel Kariuki, Firdausi Qadri, John D Clemens

**Affiliations:** International Centre for Diarrhoeal Disease Research, Bangladesh, Dhaka, Bangladesh; International Centre for Diarrhoeal Disease Research, Bangladesh, Dhaka, Bangladesh; International Centre for Diarrhoeal Disease Research, Bangladesh, Dhaka, Bangladesh; International Centre for Diarrhoeal Disease Research, Bangladesh, Dhaka, Bangladesh; Kenya Medical Research Institute (KEMRI), Nairobi, Kenya; Research Investment for Global Health Technology (RIGHT) Foundation, Seoul, Republic of Korea; International Vaccine Institute, Seoul, Republic of Korea; Kenya Medical Research Institute (KEMRI), Nairobi, Kenya; International Vaccine Institute, Seoul, Republic of Korea; Kenya Medical Research Institute (KEMRI), Nairobi, Kenya; International Centre for Diarrhoeal Disease Research, Bangladesh, Dhaka, Bangladesh; International Centre for Diarrhoeal Disease Research, Bangladesh, Dhaka, Bangladesh; International Centre for Diarrhoeal Disease Research, Bangladesh, Dhaka, Bangladesh; International Centre for Diarrhoeal Disease Research, Bangladesh, Dhaka, Bangladesh; International Vaccine Institute, Seoul, Republic of Korea; Yonsei University Graduate School of Public Health, Seoul, Republic of Korea; Armauer Hansen Research Institute, Addis Ababa, Ethiopia; Kenya Medical Research Institute (KEMRI), Nairobi, Kenya; Kenya Medical Research Institute (KEMRI), Nairobi, Kenya; International Vaccine Institute, Seoul, Republic of Korea; Kenya Medical Research Institute (KEMRI), Nairobi, Kenya; International Vaccine Institute, Seoul, Republic of Korea; ICMR—National Institute for Research in Bacterial Infections, Kolkata, India; International Centre for Diarrhoeal Disease Research, Bangladesh, Dhaka, Bangladesh; Kenya Medical Research Institute (KEMRI), Nairobi, Kenya; International Centre for Diarrhoeal Disease Research, Bangladesh, Dhaka, Bangladesh; International Vaccine Institute, Seoul, Republic of Korea; UCLA Fielding School of Public Health, Los Angeles, California, USA; Korea University School of Medicine Vaccine Innovation Center, Seoul, Republic of Korea

**Keywords:** Bangladesh, clinical features, enterotoxigenic *Escherichia coli* (ETEC), epidemiology

## Abstract

**Background:**

Enterotoxigenic *Escherichia coli* (ETEC) is a major cause of diarrheal illness, and population-based data on the incidence of clinically significant ETEC diarrhea in developing countries are limited. We provide insight into ETEC epidemiology; we followed a population-based cohort in a vaccine trial.

**Methods:**

We analyzed data from a cluster-randomized controlled trial of an oral cholera vaccine conducted in an urban slum in Dhaka, Bangladesh. The study covers 90 geographical clusters with an average population of 2988 households/cluster (baseline population 268 896). Two cohort analyses were conducted, 1 as dynamic cohort that included all subjects at vaccination, in-migrants, and births over 4 years and a closed cohort, which included only individuals present at baseline. We evaluated individuals placed under treatment center–based diarrheal surveillance between 2011 and 2015.

**Results:**

In the dynamic cohort, the ETEC incidence was 150/100 000 person-years (PY; 95% CI, 141–159), with seasonal peaks during warmer months, and in the closed cohort, the incidence was 153/100 000 PY (95% CI, 140, 166). The highest rate was seen in children aged <1 year (2007; 95% CI, 1664–2402), then in those aged 1–4 years (314; 95% CI, 252–386), and again the rate rose in those aged >45 years (219/100 000 PY; 95% CI, 177–267). The rate of severe ETEC was ≤35/100 000 PY for persons aged ≤45 years (95% CI, 27–44), but rose to 82 for adults aged >45 years (95% CI, 58–113).

**Conclusions:**

ETEC diarrhea is a major health problem in young children and older adults, prevention through vaccination and improved water, sanitation, and hygiene should target both age groups.

Enterotoxigenic *Escherichia coli* (ETEC), which causes diarrhea by secretion of heat-stable toxin (ST), heat-labile toxin (LT), or both, is a leading cause of bacterial diarrhea among children aged <5 years in developing countries. Globally, ETEC diarrhea causes 220 million cases annually [1], with 84.4 million cases reported in children aged <5 years, resulting in ∼44 400 (29 400–59 800) deaths each year [2]. Furthermore, ETEC infection significantly contributes to traveler's diarrhea [3, 4].

Approximately 40% of bacterial diarrheal infections are attributed to ETEC and/or *Vibrio cholerae* O1 in Bangladesh [5, 6]. In 2022, a snapshot of a diarrheal epidemic in Dhaka suggested that ETEC surpassed cholera as a cause of diarrhea [7]. Moreover, ETEC has been found in 14% of “point of drinking” water (at the point of consumption) and 18% of “public domain” water sources (at the public source) in urban areas of Dhaka [8].

Several studies have been conducted to measure the incidence of ETEC ascertained through active household surveillance, which primarily assesses mild diarrhea; there are limited population-based data on the incidence of clinically significant ETEC diarrhea, ascertained by surveillance of diarrhea evaluated in clinical treatment settings [2, 9]. As clinically significant ETEC diarrhea is the disease outcome of greatest relevance to the development and ultimate deployment of new-generation ETEC vaccines, this leaves an important gap in our knowledge.

A large effectiveness trial of an inactivated oral cholera vaccine (OCV) was conducted in Mirpur, an urban slum of Dhaka, and participants were followed for 4 years to detect *V. cholerae* as well as ETEC through passive surveillance [10]. This trial provided an opportunity to obtain population-based data on clinically significant ETEC diarrhea in the community. Herein, we present data on the descriptive epidemiology of clinically significant ETEC diarrhea from a large, prospectively followed population-based cohort in a vaccine field trial.

## METHODS

We analyzed data from the cluster-randomized controlled trial that investigated the effectiveness of killed whole-cell OCV consisting of O1 and O139 serogroups without B subunit cholera toxin (Shanchol, Shantha Biotech, Hyderabad, India) in combination with a water, sanitation, and hygiene (WASH) intervention that protected people from cholera infection in 6 wards of the Mirpur area in Dhaka, Bangladesh [10]. The vaccine evaluated in this study has no known protection against ETEC diarrhea. Ninety geographical clusters with an average population of 2988 households per cluster (baseline population 268 896) were randomly allocated into 3 trial arms receiving either OCV only, OCV and a WASH intervention, or no intervention. The WASH intervention included the installation of a “liquid chlorine” dispensing pump in homes for drinking water treatment and soapy water for handwashing [10].

A baseline census was carried out from April to September 2010. Verbal consent was obtained from households to obtain information on demographics, socioeconomic status (SES), and WASH practices. The census was updated twice a year until April 2015, detailing updates on events including births, deaths, and migrations. The vaccine was administered from February 17 to April 16, 2011, and 2 doses were given at 14-day intervals, targeting healthy individuals aged 1 year or older, excluding pregnant women. Passive surveillance for diarrhea was carried out in 12 health facilities, including 2 icddr,b hospitals (Dhaka Hospital, and Mirpur Treatment Center) and 10 other health facilities serving the study area [10]. These health facilities were selected based on the responses from the participants during baseline census. All patients who visited health facilities with diarrhea within 24 hours before the presentation were enrolled in the study. We analyzed the data of participants included in diarrheal surveillance between February 2011 and April 2015.

The clinical staff at each hospital were trained for diarrheal surveillance and directly responsible for dealing with the patients coming from the study sites. One surveillance staff member was stationed at each hospital to enroll diarrheal cases from the study areas and obtain clinical histories including intravenous saline consumption, oral rehydration solution intake, drug use, physical examinations, collected fecal specimens, and initial and final diagnosis on discharge.

Participants in the health facilities were identified using their study identification cards or by searching for their identities in an on-site computerized census database. Data were collected using a structured questionnaire, and fecal specimens were obtained after obtaining written informed consent or assent. Specimens were transferred to the icddr,b laboratory in Cary-Blair media for the detection of *V. cholerae* using conventional culture methods [10] and ETEC within 6 hours after collection. To detect ETEC, fresh stool specimens were plated onto MacConkey agar and incubated at 37°C for 18 hours. Six lactose-fermenting colonies with a morphology resembling ETEC were immediately tested for ETEC toxins including LT and ST enterotoxins using multiplex polymerase chain reaction (PCR) [11].

### Definitions

Diarrhea was defined as a visit where the patient reported 3 or more loose stools or 1–2 or an indeterminate number of loose stools with signs of dehydration within the last 24 hours before presentation. Dehydration was assessed following World Health Organization (WHO) guidelines [12]. Repeat diarrheal visits for an individual occurring within 7 days of discharge from the previous visit were grouped as part of the same episode. The onset of a diarrheal episode was defined as the onset of symptoms for the first diarrheal visit of an episode. An ETEC episode was defined as a nonbloody diarrheal episode where ETEC was confirmed in fecal specimens by multiplex PCR [11].

### Strategy for Analysis

We described the epidemiology of ETEC diarrhea patients seeking care in this large, prospectively followed cohort. We limited our analysis to all individuals, regardless of vaccination status, in clusters in the “OCV only” and “no intervention” arms. We performed 2 types of cohort analyses. We evaluated a dynamic cohort to capture the entire population throughout the study in this highly mobile population, thus providing the best depiction of disease burden overall, and we evaluated the cohort by age, sex, and year of follow-up. This cohort included all subjects residing in the study clusters at the time of vaccination, as well as in-migrants and births thereafter over the entire 4-year study period. Follow-up was right-censored by deaths or out-migrations. The outcomes were all ETEC episodes irrespective of diarrheal co-pathogens, including both initial and recurrent episodes. The measure of incidence was ETEC cases per person-time of follow-up, and rates by age and calendar time referred to age or calendar interval at the time of follow-up.

We also evaluated a closed cohort consisting only of persons residing in the study clusters at the time of vaccination. The purpose of this analysis was to provide estimates of ETEC incidence that may inform the deployment of future ETEC vaccines. Follow-up of individuals in both cohorts was censored by postentry death or out-migration. The outcomes were limited to the first ETEC episodes. Incidence was measured cumulatively from baseline and expressed both as rate and risk. Thus, analysis of incidence by age referred to the age at baseline, and estimates by calendar year of follow-up referred to the cumulative incidence from baseline until the end of the cited calendar interval.

### Statistical Analysis

We defined the start date of follow-up as the date of the first vaccine dose for OCV recipients or the median date of the first vaccination for nonrecipients of the vaccine in the vaccine clusters and as the median date of the first dose in the nearest vaccinated cluster for residents in the clusters not allocated to OCV. For the population entering the study area after OCV delivery in the dynamic cohort, the study start date was determined by the date of entry or birth. ETEC episodes that began on or after the start date were eligible for the analyses.

In simple estimates of incidence rates for the dynamic cohort, the number of episodes of ETEC was the numerator and the corresponding person-time of follow-up was the denominator. When estimating incidence by age or season at follow-up, episodes were those in the age or calendar-time period of interest, and the denominator was the person-time of follow-up during this period. Ninety-five percent CIs for rates were estimated using the Byar method [13]. Simple estimates of cumulative risk for the closed cohort used Kaplan-Meier curves [14]. CIs for these risks were estimated with the Greenwood formula [13].

Simple comparisons of categorical variables were conducted using the chi-square test or Fisher exact test when data were sparse, and those for continuous variables used the Student *t* test, or the Mann-Whitney *U* test when distributional assumptions for the Student *t* test were not fulfilled. Associations between household SES variables and WASH variables with ETEC incidence rates employed Cox regression models. In these models, age at baseline was forced as an independent variable, implemented after verifying that all independent variables' proportionality assumptions were met. The clustering of ETEC diarrhea in households using intraclass correlation coefficients (ICCs) and significance were tested by the Kruskal-Wallis rank-sum test. *P* values and 95% CIs were adjusted to account for the original trial design effect. For all analyses, a threshold of *P* < .05 (2-tailed) was considered statistically significant. The analyses were performed using R statistical software (version 4.2.1), employing “epiR” to estimate incidence rates and “iccCounts” for the calculation of ICCs.

## RESULTS

**Figure 1.**
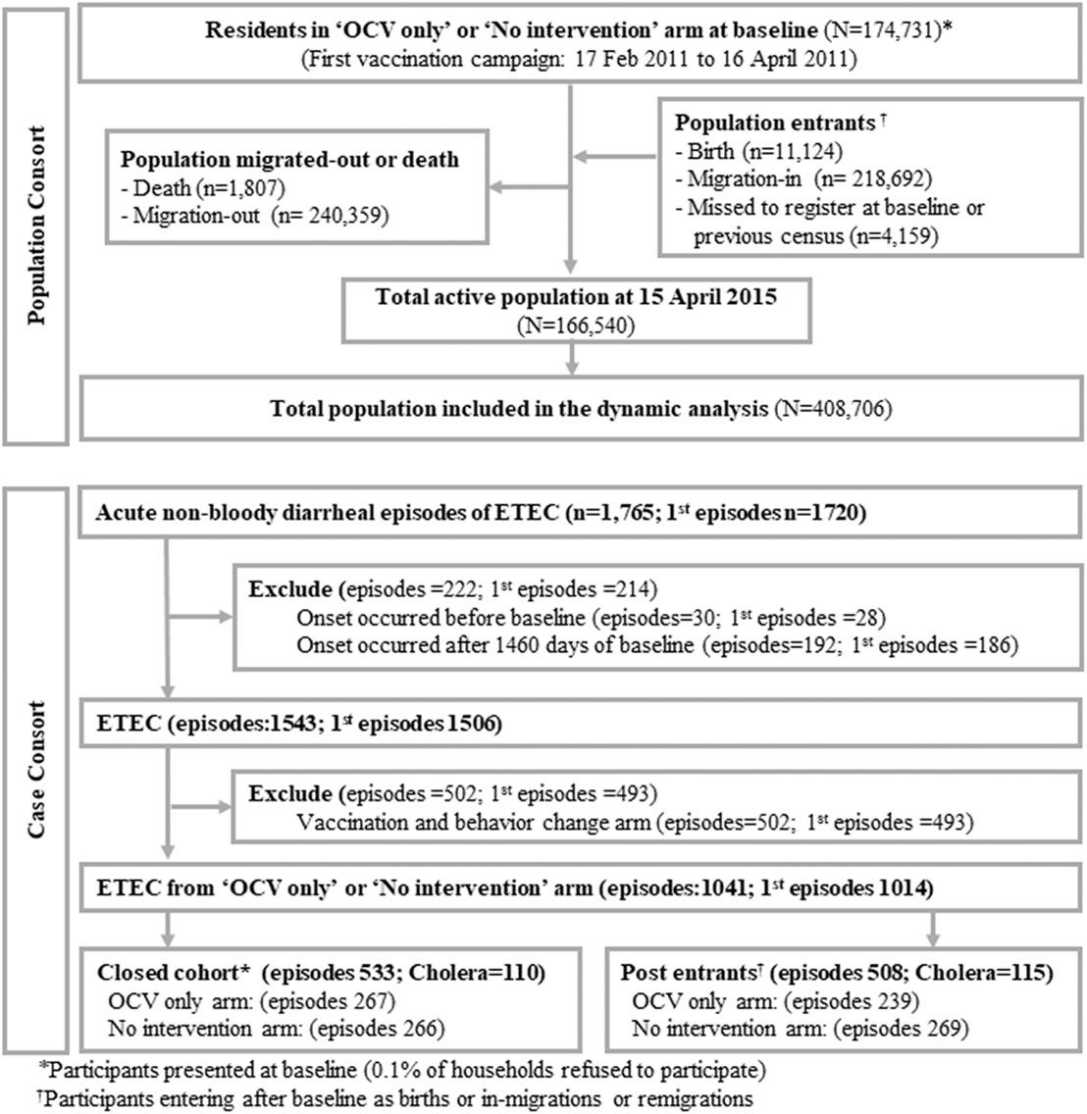
Consort diagram of the ETEC episodes. Abbreviations: ETEC, enterotoxigenic *Escherichia coli*; OCV, oral cholera vaccine.

A total of 408 706 individuals were included in the dynamic, and 174 731 individuals were included in the closed cohort analysis. The high migration rates of this population were reflected through 218 692 migrations-in and 240 359 migrations-out of the total population over 4 years follow-up. We observed 1041 episodes of ETEC diarrhea, 225 (22%) of which involved *V. cholerae* O1 coinfections. Among these episodes, 533 were from the baseline population, while the remaining 508 episodes were observed in individuals who entered the study area after baseline (Figure 1).

Of the total episodes of ETEC diarrhea, 1014 individuals experienced only 1 episode and 13 individuals had recurrent episodes (Figure 1). Among these recurrent episodes, 12 individuals had 2 episodes and 1 individual had 3 episodes of ETEC diarrhea.

### The Incidence Rate of ETEC Diarrhea by Year of Follow-up, Age, and Sex

**Figure 2.**
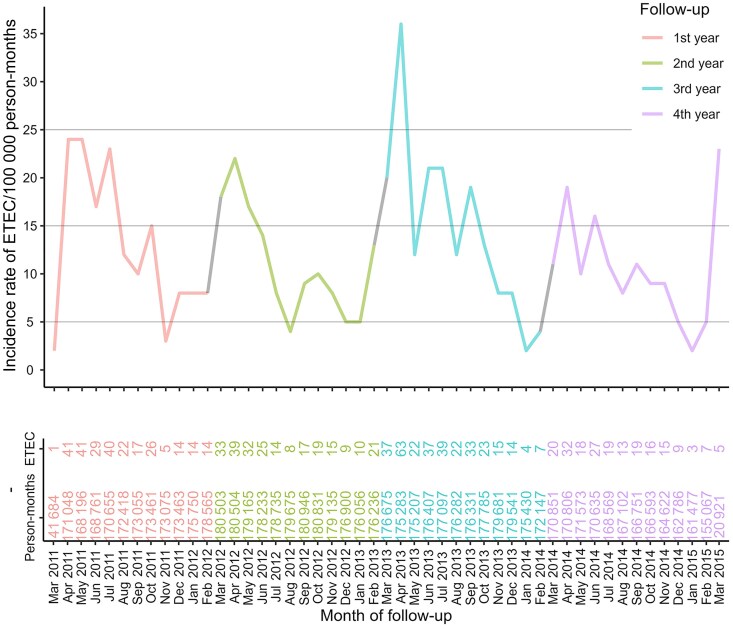
Incidence rate of ETEC diarrhea by date of follow-up in the dynamic cohort. Abbreviation: ETEC, enterotoxigenic *Escherichia coli*.

In the dynamic cohort, the overall incidence rate of ETEC diarrhea, with or without *V. cholerae* O1 coinfection, over the 4 years of follow-up was 150 episodes per 100 000 person-years (PY; 95% CI, 141–159) (Table 1). The highest incidence was observed in the third year of follow-up (219 episodes per 100 000 PY), and the point estimate of rates ranged from 148 to 219 episodes per 100 000 PY during 4 years of follow-up. Incidence rates of ETEC diarrhea were highest during premonsoon (March–May) and postmonsoon (September–November) months (Figure 2). Analysis of incidence by age at follow-up showed that the age group <1 year had the highest overall incidence rate, 1353 per 100 000 PY (95% CI, 1164–1564), followed by the 1–4 years age group, 621 per 100 000 PY (95% CI, 556–692). The incidence rate was lowest in children aged 5–14 years, 38 per 100 000 PY (95% CI, 29–50), followed by persons aged 15–45 years, 80 per 100 000 PY (95% CI, 72–89). Incidence increased in persons aged >45 years, 196 per 100 000 PY (95% CI, 168–229). The incidence rates of ETEC diarrhea among males and females were comparable throughout the follow-up period (Table 1), and patterns by age and gender were similar when ETEC episodes with *V. cholerae* O1 coinfections were excluded in the dynamic cohort analysis and when cumulative incidence was analyzed in the closed cohort (Table 2; Supplementary Figure 1).

**Table 1.** Yearly and Cumulative Occurrence of All and Severe ETEC Diarrheal Episodes, Regardless of *Vibrio cholerae* O1 Coinfection, in the Dynamic vs Closed Cohorts

Strata	Groups	Dynamic Cohort^a^							Closed Cohort^b^						
		ETEC				Severe Dehydration ETEC			ETEC				Severe Dehydration ETEC		
		No.	Cases	IR (95% CI)	*P* ^c^	Cases	IR (95% CI)	*P* ^c^	No.	Cases	CIR (95% CI)	*P* ^c^	Cases	CIR (95% CI)	*P* ^c^
Overall	All	408 706	1041	150 (141–159)		196	28 (24–32)		174 731	533	153 (140–166)		115	33 (27–39)	
	<1 y	20 728	176	1353 (1164–1564)		0	0 (0–19)		3001	114	2007 (1664–2402)		0	0 (0–43)	
	1–4 y	44 919	324	621 (556–692)		13	25 (14–41)		14 303	85	314 (252–386)		7	26 (12–51)	
	5–14 y	85 115	53	38 (29–50)	<.001	11	8 (4–14)	<.001	36 076	27	35 (24–50)	<.001	6	8 (3–16)	<.001
	15–45 y	265 744	330	80 (72–89)		109	26 (22–32)		103 622	216	110 (96–125)		68	35 (27–44)	
	>45 y	41 651	158	196 (168–229)		63	78 (61–100)		17 729	91	219 (177–267)		34	82 (58–113)	
	Female	211 373	542	153 (140–166)		124	35 (29–42)		89 804	285	163 (145–182)		72	41 (32–51)	
	Male	197 333	499	146 (134–160)	.823	72	21 (17–26)	.001	84 927	248	143 (126–162)	.339	43	25 (18–33)	.016
Year-1	All	237 823	283	174 (155–196)		43	26 (19–35)		174 731	247	181 (159–204)		41	12 (9–16)	
	<1 y	7769	75	2370 (1878–2954)		0	0 (0–78)		3001	73	3102 (2451–3877)		0	0 (0–43)	
	1–4 y	22 210	82	652 (522–805)		3	24 (7–64)		14 303	53	489 (370–634)		3	11 (3–30)	
	5–14 y	49 291	11	33 (18–58)	<.001	3	9 (3–24)	.018	36 076	11	38 (20–66)	<.001	3	4 (1–10)	.036
	15–45 y	146 318	86	90 (73–111)		30	32 (22–44)		103 622	86	108 (87–133)		28	14 (10–20)	
	>45 y	25 021	29	158 (108–224)		7	38 (17–75)		17 729	24	162 (107–237)		7	17 (7–33)	
	Female	122 663	150	181 (154–211)		28	34 (23–48)		89 804	131	188 (158–222)		26	15 (10–21)	
	Male	115 160	133	168 (141–198)	.631	15	19 (11–30)	.076	84 927	116	173 (144–207)	.606	15	9 (5–14)	.124
Year-2	All	218 579	241	157 (138–178)		37	24 (17–33)		174 731	373	107 (97–118)		65	29 (22–36)	
	<1 y	7929	38	1146 (823–1555)		0	0 (0–74)		3001	105	1849 (1520–2229)		0	0 (0–65)	
	1–4 y	19 630	102	913 (748–1103)		3	27 (7–72)		14 303	74	273 (216–341)		5	28 (11–62)	
	5–14 y	43 638	10	33 (17–58)	<.001	3	10 (3–26)	.003	36 076	18	23 (14–36)	<.001	4	8 (3–19)	.002
	15–45 y	134 605	55	61 (46–78)		19	21 (13–32)		103 622	127	65 (54–76)		42	32 (24–43)	
	>45 y	23 645	36	200 (142–273)		12	67 (36–113)		17 729	49	118 (88–154)		14	54 (31–89)	
	Female	112 343	115	147 (122–175)		25	32 (21–46)		89 804	195	111 (96–128)		42	36 (27–49)	
	Male	106 236	126	167 (140–199)	.253	12	16 (9–27)	.049	84 927	178	103 (89–119)	.733	23	21 (13–30)	.033
Year-3	All	208 473	318	219 (196–245)		78	54 (43–67)		174 731	475	161 (147–176)		99	34 (27–41)	
	<1 y	7422	38	1237 (889–1680)		0	0 (0–80)		3001	113	2327 (1927–2786)		0	0 (0–51)	
	1–4 y	18 101	95	915 (745–1114)		6	58 (24–119)		14 303	83	361 (290–445)		6	26 (11–54)	
	5–14 y	40 759	22	79 (51–118)	<.001	3	11 (3–29)	<.001	36 076	24	37 (24–54)	<.001	5	8 (3–17)	<.001
	15–45 y	129 423	115	133 (111–159)		41	48 (35–64)		103 622	179	107 (92–123)		60	36 (28–46)	
	>45 y	23 102	48	276 (206–362)		28	161 (109–229)		17 729	76	220 (175–274)		28	81 (55–116)	
	Female	106 882	176	239 (205–276)		51	69 (52–90)		89 804	257	173 (153–195)		67	45 (35–57)	
	Male	101 591	142	200 (169–235)	.146	27	38 (26–54)	.013	84 927	218	150 (131–170)	.238	32	22 (15–31)	.001
Year-4	All	187 734	199	148 (129–170)		38	28 (20–38)		…	…	…		…	…	
	<1 y	7095	25	844 (559–1225)		0	0 (0–83)		…	…	…		…	…	
	1–4 y	16 293	45	466 (345–618)		1	10 (1–48)		…	…	…		…	…	
	5–14 y	35 813	10	40 (20–71)	<.001	2	8 (2–26)	<.001	…	…	…		…	…	
	15–45 y	116 182	74	93 (73–116)		19	24 (15–36)		…	…	…		…	…	
	>45 y	21 729	45	268 (198–355)		16	95 (57–151)		…	…	…		…	…	
	Female	95 726	101	148 (122–180)		20	29 (19–45)		…	…	…		…	…	
	Male	92 008	98	148 (121–180)	.947	18	27 (17–42)	.840	…	…	…		…	…	

Abbreviations: CIR, cumulative incidence rate/100 000 person-years; ETEC, enterotoxigenic *Escherichia coli*; IR, incidence rate/100 000 person-years.

^a^Dynamic cohort: “Age group” refers to the age at infection, while “person-time” represents the exact follow-up time spent within specific age groups. The overall incidence rates are calculated for all 4 years combined—year 1: start date to <1 year; year 2: 1 to <2 years; year 3: 2 to <3 years; and year 4: 3 to 4 years of follow-up.

^b^Closed cohort: “Age group” refers to age at baseline. The overall incidence rates are calculated for all 4 years combined—year 1: baseline to <1 year; year 2: baseline to <2 years; year 3: baseline to 3 years.

^c^ *P* value calculated using the Cochran-Armitage test for trend.

**Table 2.** Yearly and Cumulative Occurrence of All and Severe ETEC Diarrheal Episodes, Excluding ETEC Episodes With *Vibrio cholerae* O1 Coinfection, in the Dynamic vs Closed Cohorts

Strata	Groups	Dynamic Cohort^a^							Closed Cohort^b^						
		ETEC				Severe Dehydration ETEC			ETEC				Severe Dehydration ETEC		
		No.	Cases	IR (95% CI)	*P* ^c^	Cases	IR (95% CI)	*P* ^c^	No.	Cases	CIR (95% CI)	*P* ^c^	Cases	CIR (95% CI)	*P* ^c^
Overall	All	408 706	816	117 (109–126)		103	15 (12–18)		174 731	423	121 (110–133)		68	20 (15–25)	
	<1 y	20 728	151	1161 (987–1357)		0	0 (0–19)		3001	98	1726 (1409–2093)		0	0 (0–43)	
	1–4 y	44 919	266	510 (451–574)		2	4 (1–12)		14 303	66	244 (190–308)		1	4 (0–17)	
	5–14 y	85 115	38	28 (20–37)	<.001	5	4 (1–8)	<.001	36 076	22	29 (18–43)	<.001	5	6 (2–14)	<.001
	15–45 y	265 744	246	60 (53–68)		62	15 (12–19)		103 622	167	85 (73–98)		41	21 (15–28)	
	>45 y	41 651	115	143 (119–171)		34	42 (30–58)		17 729	70	168 (132–211)		21	50 (32–76)	
	Female	211 373	415	117 (106–129)		67	19 (15–24)		89 804	223	127 (111–145)		44	25 (18–33)	
	Male	197 333	401	118 (107–130)	.623	36	11 (8–14)	.007	84 927	200	116 (100–133)	.587	24	14 (9–20)	.028
Year 1	All	237 823	239	147 (129–167)		30	18 (13–26)		174 731	206	151 (131–173)		28	8 (5–11)	
	<1 y	7769	67	2118 (1655–2672)		0	0 (0–78)		3001	64	2720 (2113–3450)		0	0 (0–43)	
	1–4 y	22 210	68	541 (423–681)		1	8 (1–37)		14 303	43	397 (291–529)		1	4 (0–17)	
	5–14 y	49 291	10	30 (16–54)	<.001	3	9 (3–24)	.024	36 076	10	35 (18–61)	<.001	3	4 (1–10)	.041
	15–45 y	146 318	69	72 (57–91)		21	22 (14–33)		103 622	69	87 (68–109)		19	10 (6–15)	
	>45 y	25 021	25	136 (90–198)		5	27 (10–60)		17 729	20	135 (85–205)		5	12 (5–26)	
	Female	122 663	127	153 (128–181)		21	25 (16–38)		89 804	109	157 (129–188)		19	11 (7–17)	
	Male	115 160	112	141 (117–169)	.629	9	11 (6–21)	.044	84 927	97	145 (118–176)	.663	9	5 (3–10)	.081
Year 2	All	218 579	203	132 (115–151)		25	16 (11–24)		174 731	307	88 (79–98)		45	20 (15–26)	
	<1 y	7929	34	1025 (722–1415)		0	0 (0–74)		3001	90	1585 (1282–1938)		0	0 (0–65)	
	1–4 y	19 630	88	787 (635–965)		1	9 (1–42)		14 303	60	221 (171–283)		1	6 (1–26)	
	5–14 y	43 638	7	23 (10–45)	<.001	1	3 (0–15)	<.001	36 076	15	19 (11–31)	<.001	4	8 (3–19)	<.001
	15–45 y	134 605	42	46 (34–62)		12	13 (7–22)		103 622	100	51 (42–62)		28	21 (15–31)	
	>45 y	23 645	32	177 (124–247)		11	61 (32–106)		17 729	42	101 (74–135)		12	47 (25–79)	
	Female	112 343	89	113 (92–139)		15	19 (11–31)		89 804	156	89 (76–104)		29	25 (17–36)	
	Male	106 236	114	151 (126–181)	.031	10	13 (7–24)	.389	84 927	151	87 (74–102)	.839	16	14 (9–23)	.080
Year 3	All	208 473	235	162 (142–184)		35	24 (17–33)		174 731	382	130 (117–143)		62	21 (16–27)	
	<1 y	7422	33	1075 (753–1490)		0	0 (0–80)		3001	97	1998 (1629–2426)		0	0 (0–51)	
	1–4 y	18 101	74	713 (564–890)		0	0 (0–24)		14 303	65	283 (220–358)		1	4 (0–20)	
	5–14 y	40 759	16	58 (34–91)	<.001	1	4 (0–17)	<.001	36 076	19	29 (18–45)	.001	4	6 (2–15)	<.001
	15–45 y	129 423	84	97 (78–120)		23	27 (17–39)		103 622	142	85 (72–99)		39	23 (17–31)	
	>45 y	23 102	28	161 (109–229)		11	63 (34–109)		17 729	59	171 (132–219)		18	52 (32–81)	
	Female	106 882	128	174 (145–206)		23	31 (20–46)		89 804	201	135 (117–155)		41	28 (20–37)	
	Male	101 591	107	150 (124–181)	.327	12	17 (9–29)	.087	84 927	181	124 (107–143)	.633	21	14 (9–22)	.020
Year 4	All	187 734	139	104 (87–122)		13	10 (5–16)		…	…	…		…	**…**	
	<1 y	7095	17	574 (347–898)		0	0 (0–83)		…	…	…		…	**…**	
	1–4 y	16 293	36	373 (266–511)		0	0 (0–26)		…	…	…		…	**…**	
	5–14 y	35 813	5	20 (8–44)	<.001	0	0 (0–10)	<.001	…	…	…		…	**…**	
	15–45 y	116 182	51	64 (48–83)		6	8 (3–16)		…	…	…		…	**…**	
	>45 y	21 729	30	179 (123–252)		7	42 (19–82)		…	…	…		…	**…**	
	Female	95 726	71	104 (82–131)		8	12 (6–22)		…	…	…		…	**…**	
	Male	92 008	68	103 (80–129)	.983	5	8 (3–17)	.447	…	…	…		…	**…**	

Abbreviations: CIR, cumulative incidence rate/100 000 person-years; ETEC, enterotoxigenic *Escherichia coli*; IR, incidence rate/100 000 person-years.

^a^Dynamic cohort: “Age group refers” to the age at infection, while “person-time” represents the exact follow-up time spent within specific age groups. The overall incidence rates are calculated for all 4 years combined—year 1: start date to <1 year; year 2: 1 to <2 years; year 3: 2 to <3 years; and year 4: 3 to 4 years of follow-up.

^b^Closed cohort: “Age group” refers to age at baseline. Overall incidence rates are calculated for all 4 years combined—year 1: baseline to <1 year; year 2: baseline to <2 years; year 3: baseline to 3 years.

^c^ *P* value calculated using the Cochran-Armitage test for trend.

In the dynamic cohort, the incidence rate of ETEC diarrhea with severe dehydration (28 episodes per 100 000 PY; 95% CI, 24–32) was ∼18.8% (196/1041), as high as the incidence of all ETEC diarrhea (Table 1). In contrast to the assessment of all ETEC episodes in the overall analysis, rates of severe ETEC diarrhea were modest (<26 episodes per 100 000 PY) in all younger age groups (<15 years), rising to 78 per 100 000 PY (95% CI, 61–100) in those aged >45 years. Also in contrast to the analysis of all ETEC episodes, rates of severe ETEC diarrhea in the overall analysis were higher in females (35 episodes per 100 000 PY; 95% CI, 29–42) than in males (21 episodes per 100 000 PY; 95% CI, 17–26) (Table 1). Similar patterns of incidence rates of severe ETEC diarrhea were seen when ETEC episodes with *V. cholerae* O1 coinfections were excluded in the dynamic cohort analysis and when cumulative incidence was analyzed in the closed cohort (Table 2). When we measured the risk of ETEC based on baseline participants using the Kaplan-Meier curve, the risk of severe dehydration was significantly higher among individuals aged >45 years during the 4-year follow-up (Supplementary Figure 2).

### Household Socioeconomic Factors Related to the Occurrence of ETEC in the Closed Cohort

The incidence rate of ETEC was 96 per 100 000 PY (95% CI, 80–115) among individuals residing in their own houses, a marker of higher SES, a rate that was 20% lower than that for individuals living in a rental house (hazard ratio [HR], 0.80; 95% CI, 0.64–0.99; *P* = .038). Similarly, individuals living in households with more than 1 room, also a marker of higher SES, had a significantly lower incidence of ETEC than those who lived in homes with 1 room (HR, 0.76; 95% CI, 0.6–0.98; *P* = .031). No significant association was observed between ETEC incidence and living in a home with a shared toilet or kitchen, having a fixed waste disposal place, having family members who washed their hands with soap, or using a water filter (Table 3). Additionally, no clustering of ETEC was observed at the household level (ICC < 0.001; *P* = .499); we did not consider household clustering as a design effect in the models.

**Table 3.** The Association of Household Socioeconomic Features Status at Baseline and Occurrence of ETEC Diarrhea, Excluding Episodes With Cholera Coinfection, in the Closed Cohort

	Participants	PY	Case	IR (95% CI)	HR^a^	*P* ^b^
Monthly expenditure (BDT)						
<7900	60 208	112 835	142	126 (106–148)	Ref	-
7900 to <9840	40 927	80 266	96	120 (97–145)	0.95 (0.73–1.24)	.710
9840 to <12 000	35 922	68 867	81	118 (94–145)	0.94 (0.7–1.26)	.659
≥12 000	37 674	86 213	104	121 (99–146)	0.99 (0.80–1.23)	.953
House ownership type						
Rent house	134 755	222 595	302	136 (121–152)	Ref	-
Own house	39 976	125 587	121	96 (80–115)	0.8 (0.64–0.99)	.038
No. of rooms in house						
1	142 829	263 867	345	131 (117–145)	Ref	-
>1	31 902	84 314	78	93 (74–115)	0.76 (0.6–0.98)	.031
House floor made of brick/cement						
No	16 858	35 272	35	99 (70–136)	Ref	-
Yes	157 873	312 910	388	124 (112–137)	1.24 (0.83–1.85)	.292
Shared kitchen						
No	26 004	71 089	69	97 (76–122)	Ref	-
Yes	148 727	277 092	354	128 (115–142)	1.23 (0.90–1.66)	.188
Shared toilet						
No	9631	26 216	24	92 (60–134)	Ref	-
Yes	165 100	321 965	399	124 (112–137)	1.27 (0.86–1.87)	.232
Waste disposal place						
No	36 644	78 301	88	112 (91–138)	Ref	-
Yes	138 087	269 880	335	124 (111–138)	1.09 (0.77–1.55)	.610
Use of water filter						
No	172 243	342 074	417	122 (111–134)	Ref	-
Yes	2488	6107	6	98 (41–203)	0.84 (0.39–1.78)	.643
Hand wash using soap						
No	16 087	30 134	36	119 (85–163)	Ref	-
Yes	158 644	318 048	387	122 (110–134)	1.03 (0.72–1.49)	.860

Abbreviations: BDT, Bangladeshi taka (1 BDT = 0.0085USD); ETEC, enterotoxigenic *Escherichia coli*; HR, hazard ratio; IR, incidence rate/100 000 person-years.

^a^Hazard ratio adjusted by age.

^b^ *P* value calculated using Cox regression model.

### Clinical and Laboratory Characteristics of ETEC Diarrhea in a Dynamic Cohort

Overall, 12.6% (103/816) of episodes of ETEC diarrhea without *V. choleare* coinfection presented with severe dehydration, and 41.8% (341 episodes) with some sign of dehydration. Among the 417 ETEC episodes in children aged <5 years, 0.5% (2 episodes) presented with severe dehydration; in the age group 5–14 years (out of 38 episodes, 5 episodes), 13.2% experienced severe signs of dehydration, and among individuals aged 15 years and older, 26.6% (out of 361 episodes, 96 episodes) presented with severe signs of dehydration (Table 4). Older age at presentation was highly associated with a greater level of dehydration at presentation (*P* < .001), a finding that mirrored population-based incidence (Tables 1 and 2). ETEC with *V. cholera* coinfection showed similar findings (Supplementary Table 1).

**Table 4.** Distribution of ETEC Episodes by Severity, Toxin Phenotype, and Age at Presentation in the Dynamic Cohort

Dehydration Status	Overall	<5 Years	5–14 Years	15+ Years	*P* ^a^
No sign	372 (45.6)	325 (77.9)	11 (28.9)	36 (10.0)	<.001
Some sign	341 (41.8)	90 (21.6)	22 (57.9)	229 (63.4)	
Severe sign	103 (12.6)	2 (0.5)	5 (13.2)	96 (26.6)	
Toxin phenotype					
LT	315 (38.6)	181 (43.4)	17 (44.7)	117 (32.4)	.009
ST	242 (29.7)	122 (29.3)	7 (18.4)	113 (31.3)	
LT-ST	259 (31.7)	114 (27.3)	14 (36.8)	131 (36.3)	
Total	816	417	38	361	

Two hundred twenty-five ETEC cases exc*l*uded due to cholera coinfection.

Abbreviations: ETEC, enterotoxigenic *Escherichia coli*; LT, heat-labile toxin; ST, heat-stable toxin.

^a^ *P* value calculated using chi-square test.

Among all episodes, 38.6% (315 episodes) were associated with LT-only ETEC, 31.7% (259 episodes) with LT-ST ETEC, and 29.7% (242 episodes) with ST-only ETEC. The distribution of episodes by ETEC toxin phenotype varied significantly across age groups (*P* = .009). LT-only ETEC was the most common in children aged <5 years (43.4%) and in older children aged 5–14 years (44.7%). In contrast, roughly equal proportions of episodes in children aged 15 years and older were due to each of the 3 toxin phenotypes (Table 4). There was an association (*P* = .048) between ETEC toxin phenotype and severity, with a higher proportion (51.4%, 162 episodes) of ST-only ETEC presenting no signs of dehydration (Table 5). ETEC with *V. cholera* coinfection showed similar findings (Supplementary Table 2).

**Table 5.** Distribution of ETEC Episodes by Toxin Phenotype and Severity of Dehydration in the Dynamic Cohort

Dehydration	Overall	LT	ST	LT-ST	*P* ^a^
No sign	372 (45.6)	102 (39.4)	162 (51.4)	108 (44.6)	.048
Some sign	341 (41.8)	120 (46.3)	114 (36.2)	107 (44.2)	
Severe sign	103 (12.6)	37 (14.3)	39 (12.4)	27 (11.2)	
Total	816	259	315	242	

Two hundred twenty-five ETEC cases excluded due to cholera coinfection.

Abbreviations: ETEC, enterotoxigenic *Escherichia coli*; LT, heat-labile toxin; ST, heat-stable toxin.

^a^ *P* value calculated using chi-square test.

Mortality among ETEC cases was rare; 4 deaths occurred among 1041 ETEC episodes over a 90-day follow-up period after presentation to health care facilities. Among the deceased, 3 were adults and 1 was an infant. The deaths occurred from 18 to 53 days after presentation. The patient who died 18 days after presentation with severe dehydration was a female, 43 years old, and the death occurred after discharge from the hospital.

## DISCUSSION

The incidence of ETEC diarrhea severe enough to warrant seeking clinical care was high in each of the 4 years of the study. The incidence of all episodes was highest in infants, then 1–4-year-olds, was low in older children and young adults, and then rose substantially in adults >45 years of age. The pattern of severe ETEC diarrhea differed from that for all ETEC diarrhea in that rates of severe ETEC diarrhea were low in all age groups until age 45 years, at which point they rose substantially. Reassuringly, patients who presented for care had mortality rates of <1% over the 90 days after presentation, attesting to the delivery of effective treatment. As reported from earlier studies in Bangladesh, ETEC incidence rose during the warmer and wetter months of the year [3, 6, 15]. The overall prevalence of the 3 toxin phenotypes among ETEC cases was roughly equivalent, though the distributions appeared to differ by age and by the level of dehydration of the ETEC episode.

Before discussing the implications of these findings, it is important to point out several limitations of our study. First, we did not test the stool for rotavirus and other diarrheagenic *E. coli*, so we could not separately analyze ETEC diarrheal episodes with and without rotavirus coinfections. Such coinfections might have influenced the severity of ETEC episodes, especially those occurring under 2 years old [7]. Second, our use of conventional PCR, not real-time PCR, to diagnose ETEC did not allow us to limit diagnosis of ETEC to patients with heavy fecal shedding of ETEC, thus preventing us from focusing on those diarrheal episodes caused by the isolated ETEC, rather than on all diarrhea episodes associated with the isolated ETEC, including some in which diarrheal symptoms were not due to the isolated ETEC [11]. Third, we did not characterize ETEC isolates according to colonization factors (CFs), which are important antigenic targets of many ETEC vaccines in development [16]. Lastly, there was no control group without diarrhea in this study, and we lacked data on the cause of death for the 4 deaths reported in this analysis. Despite these limitations, the study had several strengths, including its prospective, population-based approach for evaluating disease incidence and its comprehensive surveillance of the catchment area population.

We revealed that the incidence of ETEC diarrhea was higher among young children and older adults and the incidence of ETEC diarrhea with severe dehydration was high only in the elderly. Similar to our findings, an observational, health facility–based surveillance study of ETEC diarrhea in rural areas in Bangladesh revealed that children <5 years of age and adults faced a higher risk of ETEC diarrhea than older children and younger adults [17]. The peaks of rates of all detected ETEC diarrhea episodes in young children and the elderly in our study could reflect lower levels of natural anti-ETEC immunity in both groups, with waning immunity and accompanying immune senescence in the older age group [3]. On the other hand, rates of severe ETEC diarrhea in our study peaked only in the elderly. The pattern is unlikely to be a reflection of age-related health care–seeking behaviors, as parents in this setting are quick to seek care for their children if severe dehydration develops. Moreover, the difference is not likely an artifact of our surveillance, as our surveillance enrolled all known treatment centers offering care to the catchment population, and treatment sites were close to residences. The most likely explanation is the widespread use in this setting of oral rehydration solution to treat children with diarrhea at home as soon as symptoms set in [18].

Higher SES was associated with a lower risk of ETEC diarrhea, perhaps due to better WASH in the more affluent households [19]. ETEC incidence rises during the warmer months, when environmental bacterial growth rates increase, and during rainy seasons, when surface water becomes heavily contaminated with feces, enhancing disease spread [15]. Understanding the seasonality of ETEC will help policy-makers to plan targeted preventive measures such as vaccination campaigns, enhanced sanitation protocols, and food safety initiatives. The distribution of episodes by ETEC toxin varied significantly across age groups. LT-only ETEC was the most common in children under 5 years and in older children aged 5–14 years, whereas the Global Enteric Multicenter Study (GEMS) study showed that children <5 years suffered more from LT-ST and ST-toxin-type ETEC diarrhea [20]. This variation in different geographical locations may be due to genetic coding, inoculum, and geographical behavior; furthermore, in different study designs and community settings, strains may produce different levels of toxin and disease severity [21]. That LT-only ETEC was less often associated with severe dehydration in our study accords with other studies [7], though the overall prevalence associated with each of the 3 toxin phenotypes was roughly equal, underscoring the need for future ETEC vaccines to provide broad coverage against different circulating ETEC strains.

Our findings highlight the need to consider both young children and the elderly when developing, evaluating, and ultimately targeting new-generation ETEC vaccines for endemic areas. This is in contrast to current WHO recommendations focusing only on children 6–24 months of age as a target population for future ETEC vaccines [22, 23]. The high incidence of ETEC also illustrates the substantial risk of ETEC to travelers visiting endemic countries. Our findings provide estimates of ETEC incidence that will be useful for designing future phase III efficacy trials of new vaccines and suggest that incidence rates are high enough for these trials to evaluate protection against clinically significant ETEC diarrhea with feasible sample sizes.

## Supplementary Material

ofaf375_Supplementary_Data

## Supplementary Data

Supplementary materials are available at *Open Forum Infectious Diseases* online. Consisting of data provided by the authors to benefit the reader, the posted materials are not copyedited and are the sole responsibility of the authors, so questions or comments should be addressed to the corresponding author.
