# Supplementary material for: Epidemiological and Clinical Features of Enterotoxigenic Escherichia coli (ETEC) Diarrhea in an Urban Slum in Dhaka, Bangladesh
Source: Open Forum Infect Dis. 2025 Jun 30;12(7):ofaf375. doi: 10.1093/ofid/ofaf375 (PMC12272338; doi:10.1093/ofid/ofaf375)
Supplement: ofaf375_Supplementary_Data [file ofaf375_supplementary_data.zip › Supplemental table 1.docx]

**Supplementary Table 1:** **Distribution of ETEC episodes, irrespective of cholera coinfection, by severity, toxin phenotype, and age at presentation in the dynamic cohort**

| **Dehydration status** | **Overall** | **<5, years** | **5-14, years** | **15+, years** | ***P***^*^ |
| --- | --- | --- | --- | --- | --- |
| *No sign* | 432 (41.5) | 377(75.4) | 15(28.3) | 40(8.2) | <0.001 |
| *Some sign* | 413 (39.7) | 110(22.0) | 27(50.9) | 276(56.6) |  |
| *Severe sign* | 196 (18.8) | 13(2.6) | 11(20.8) | 172(35.2) |  |
| **Toxin phenotype** |  |  |  |  |  |
| *LT* | 393 (37.8) | 214(42.8) | 23(43.4) | 156(32.0) | 0.003 |
| *ST* | 319 (30.6) | 149(29.8) | 11(20.8) | 159(32.5) |  |
| *LT-ST* | 329 (31.6) | 137(27.4) | 19(35.8) | 173(35.5) |  |
| **Total** | **1041** | **500** | **53** | **488** |  |

^*^*P*-value calculated using Chi-squared
