# Supplementary material for: Epidemiological and Clinical Features of Enterotoxigenic Escherichia coli (ETEC) Diarrhea in an Urban Slum in Dhaka, Bangladesh
Source: Open Forum Infect Dis. 2025 Jun 30;12(7):ofaf375. doi: 10.1093/ofid/ofaf375 (PMC12272338; doi:10.1093/ofid/ofaf375)
Supplement: ofaf375_Supplementary_Data [file ofaf375_supplementary_data.zip › Supplemental table 2.docx]

**Supplementary Table 2: Distribution of ETEC episodes, irrespective of cholera coinfection, by toxin phenotype and severity of dehydration in the dynamic cohort**

| **Dehydration** | **Overall** | **LT** | **ST** | **LT-ST** | ***P***^*^ |
| --- | --- | --- | --- | --- | --- |
| *No sign* | 432 (41.5) | 185(47.1)^*^ | 124(38.9) | 123(37.4) | 0.061 |
| *Some sign* | 413 (39.7) | 143(36.4) | 135(42.3) | 135(41.0) |  |
| *Severe sign* | 196 (18.8) | 65(16.5) | 60(18.8) | 71(21.6) |  |
| **Total** | **1041** | **393** | **319** | **329** |  |

^*^*P*-value calculated using Chi-squared
