# Supplementary material for: Epidemiological and Clinical Features of Enterotoxigenic Escherichia coli (ETEC) Diarrhea in an Urban Slum in Dhaka, Bangladesh
Source: Open Forum Infect Dis. 2025 Jun 30;12(7):ofaf375. doi: 10.1093/ofid/ofaf375 (PMC12272338; doi:10.1093/ofid/ofaf375)
Supplement: ofaf375_Supplementary_Data [file ofaf375_supplementary_data.zip › Supplementary material legends.docx]

**Supplementary material legends**

Supplementary Figure 1: Kaplan-Meier curve of occurrence of ETEC diarrheal episodes, excluding those with V. cholerae O1 coinfection stratified by age at baseline in closed cohorts

Supplementary Figure 2: Kaplan-Meier curve of occurrence of ETEC diarrheal episodes with severe dehydration, excluding those with V. cholerae O1 coinfection, stratified by age at baseline in closed cohorts

Supplementary Table 1: Distribution of ETEC episodes, irrespective of cholera coinfection, by severity, toxin phenotype, and age at presentation in the dynamic cohort

Supplementary Table 2: Distribution of ETEC episodes, irrespective of cholera coinfection, by toxin phenotype and severity of dehydration in the dynamic cohort
