# Supplementary figures and images for: Epidemiological and Clinical Features of Enterotoxigenic Escherichia coli (ETEC) Diarrhea in an Urban Slum in Dhaka, Bangladesh
Source: Open Forum Infect Dis. 2025 Jun 30;12(7):ofaf375. doi: 10.1093/ofid/ofaf375 (PMC12272338; doi:10.1093/ofid/ofaf375)

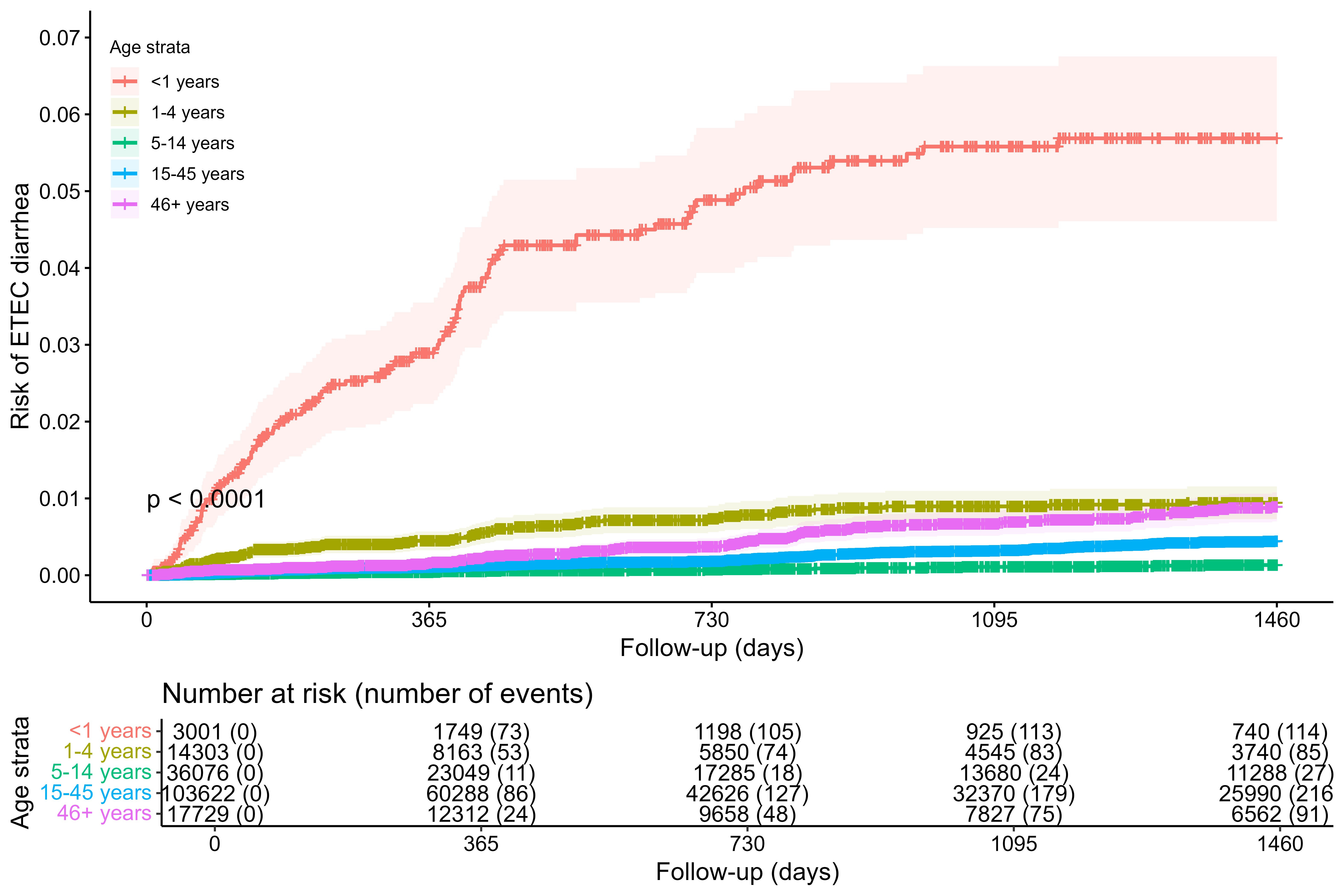

Supplement: ofaf375_Supplementary_Data [file ofaf375_supplementary_data.zip › Supplemental Figure 1 (400dpi).jpg]

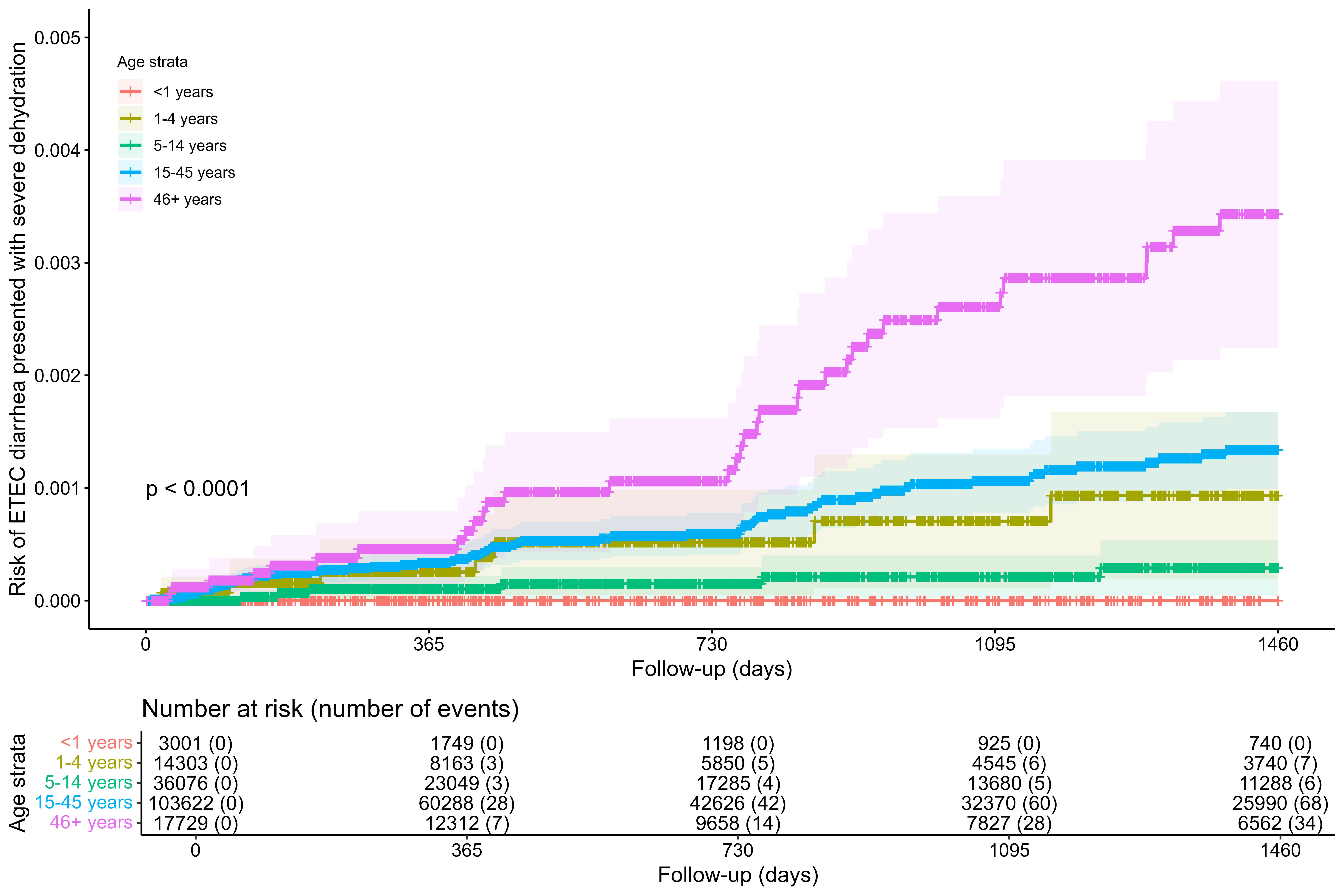

Supplement: ofaf375_Supplementary_Data [file ofaf375_supplementary_data.zip › Supplemental Figure 2 (400dpi).jpg]
